# Current epidemiological profile and features of visceral leishmaniasis in people's republic of China

**DOI:** 10.1186/1756-3305-5-31

**Published:** 2012-02-09

**Authors:** Jun-Yun Wang, Gang Cui, Hai-Tang Chen, Xiao-Nong Zhou, Chun-Hua Gao, Yue-Tao Yang

**Affiliations:** 1 National Institute of Parasitic Diseases, Chinese Center for Disease Control and Prevention; the Key Laboratory of Parasite and Vector Biology of the Chinese Ministry of Health; WHO Collaborating Center for Malaria, Schistosomiasis and Filariasis, Shanghai 200025, People's Republic of China; 2 Xinjiang Kizilsu Kirgiz Institute of Endemic disease prevention, Artux, 845350, People's Republic of China

## Abstract

**Background:**

Visceral leishmaniasis (VL) is still an important public health problem in China. In recent years endemic regions spread, prevalence increased, and even an outbreak of the disease occurred in China due to global warming and population movement. It is essential to elucidate the current epidemic situation and epidemiological characteristics of VL for designing control policy. In the present study we describe the current epidemiological profile and characteristics of VL in China based on retrospectively reviewing of VL cases reported between 2005 and 2010 by a passive surveillance system.

**Methods:**

The present study was a retrospective review of VL cases notified between 2005 and 2010 based on the passive surveillance data. The data were tabulated, diagrammatized and analyzed through descriptive statistics in a Microsoft Excel spreadsheet.

**Results:**

A total of 2450 VL cases were notified, with a mean of 408 cases per year. 61 counties were identified as endemic area with 2224 autochthonous cases, and the other 118 counties as non-endemic areas with 226 imported cases. 97.71% of cases were concentrated in Xinjiang, Gansu and Sichuan Provinces. 9 major counties reported a mean of > 10 cases per year, with a total of 1759 cases reported. Different types of VL revealed distinct epidemiological characteristics.

**Conclusions:**

The number of VL cases and endemic counties both increased in the period 2005-2010 in China. Different type or sub-type of VL revealed distinct epidemiological characteristics. Therefore, differential control measures must be taken in different endemic areas against incidence increase and endemic area spread.

## Background

Leishmaniasis is a mammalian disease caused by parasitic protozoans classified as *Leishmania *species (Kinetoplastida, Trypanosomatidae) [1-3]. The disease has different clinical forms, ranging from a skin ulcer (cutaneous leishmaniasis, CL), which can heal spontaneously, to the most severe form of visceral leishmaniasis, which can lead to the patient's death when untreated [4]. Natural transmission may be zoonotic or anthroponotic by the bite of a phlebotomine sandfly species of the genera *Phlebotomus *(Old World) and *Lutzomyia *(New World) [3,5-8].

Leishmaniasis is endemic in 98 countries or territories, with more than 350 million people at risk. The global incidence is estimated to be 2 million new cases per year (0.5 million of VL and l.5 million of CL). VL causes an estimated 50,000 deaths annually, a rate surpassed among parasitic diseases only by malaria, and 2,357,000 disability-adjusted life years lost, placing leishmaniasis ninth in a global analysis of infectious diseases [9]. Desjeux [8] indicated that in the previous decade endemic regions had spread, prevalence had increased and the number of unrecorded cases must have been substantial, because notification was compulsory in only few countries [10]. Therefore, the public health impact of the disease worldwide has been grossly underestimated [1,10].

VL has been and is still an important public health problem in China. Prior to the initiation of a national control program in 1951, VL was one of the major parasitic diseases in the People's Republic of China, endemic in 17 provinces, municipalities and autonomous regions. According to the available epidemiological data, there were about 530,000 VL cases in China in 1951 [11]. Following great efforts in the frame of the national control program, the disease has been largely brought under control in the eastern regions of the country. However, the disease is still endemic or occurs sporadically in 43 counties in six provinces or autonomous regions in western China (namely Xinjiang, Gansu, Sichuan, Shaanxi, Shanxi and Inner Mongolia) in the 1990s [12-15].

Two epidemiological types of VL can be distinguished in western China based on infected *Leishmania *species and source of infection [13,16]. The first one is an anthroponotic type of VL (AVL) caused by *L. donovani*, and currently is endemic in the oases of the plains of Kashi prefecture, Xinjiang Uygur Autonomous Region. Most cases occur in young people and adults. The transmitting cycle is from human to human and no animal host has been found (0-0.3%) [11]. The widely distributed peridomestic *Phlebotomus longiductus*, an endemic species in Xinjiang, is the vector [17].

The second one is zoonotic type caused by *L. infantum *with an animal host as the principal source of infection. This type has been divided into two subtypes, namely a mountainous and a desert sub-type based on the ecosystem and epidemiological characteristics, i.e. geographical and landscape characteristics, age distribution of patients, vector sandfly species and their ecology, and source of infection [13,16,18,19]. The mountainous sub-type of zoonotic VL (MST-ZVL) occurs in the western mountainous and hilly regions of Gansu, Sichuan, Shaanxi and Shanxi provinces. Patients are mostly children less than ten years old. High *Leishmania *spp. infection rates in dogs were detected [20,21] and elimination and prohibition of dogs markedly reduced the number of human cases, thus dogs are likely to be the principal source of infection for the subtype [13]. The vector of this form is wild *P. chinensis*, a zoophilic species that also feeds on man. During daytime, these sandflies rest mainly in caves and other shelters but they enter villages after sunset [17]. The desert sub-type of zoonotic VL (DST-ZVL) is endemic in the northwestern desert regions of China, including Xinjiang, western Inner Mongolia and northern Gansu [19]. Patients are mostly infants less than one year old [16]. These regions were uncultivated deserts before they were populated by immigrants who introduced agricultural activities. Consequently, autochthonous infantile VL occurs, and the region is considered to be a natural nidus of kala-azar infected wild animals presumably being the source of infection. However, which animals are a source of infection are unknown [16]. The wild species, *P. wui *and *P. alexandri*, are the vectors infesting the specific landscapes, such as dry desert region or the stony desert, respectively [22].

Since the disease in major endemic areas was under control with a nationwide control campaign implemented in 1950s it became neglected in China and no further surveillance and control measures was applied. In recent years endemic regions spread, prevalence increased, and even an outbreak of the disease occurred in China due to global warming and population movement [16,23,24]. It is essential to elucidate the current epidemic situation and epidemiological characteristics of VL for designing control policy. However, there was not any national VL case reporting system or surveillance system before 2005 in China, VL case records and statistics were not available, and the information on accurate number of cases, geographic distribution etc., was not clear. From 2005, VL cases must be reported compulsorily in China according to the National Regulation on the Control of Communicable Diseases. In the present study we describe the current epidemiological profile and characteristics of VL based on reported cases between 2005 and 2010.

## Methods

The present study was a retrospective review of VL cases notified between 2005 and 2010 based on the passive surveillance data reported through the web-based National Diseases Reporting Information System (NDRIS) operated by the Chinese Center for Disease Control and Prevention. In China, VL cases must be compulsorily reported through NDRIS according to the National Regulation on the Control of Communicable Diseases from 2005 [25]. All reported VL cases were diagnosed in accordance with the National Criteria for Visceral Leishmaniasis Diagnosis in China and reported via NDRIS. Passive surveillance data covered personal and clinical information, including patient's name, age, gender, address and diagnosis etc. In this study the spatial, temporal, age, occupation and gender distribution of cases were analyzed. Data were tabulated, diagrammatized and analyzed through descriptive statistics in a Microsoft Excel spreadsheet, release 2003.

The study had been reviewed and approved by the Ethical Review Committee of the National Institute of Parasitic Diseases, Chinese Center for Disease Control and Prevention in Shanghai.

## Results

### General status

Between 2005 and 2010 a total of 2450 VL cases were notified in China through NDRIS (Table 1). The yearly number of cases reported ranged from 287 to 539, with a mean of reported per year of 408 cases. In the period of 2008 to 2009 the highest number of cases was reported due to the outbreak of the disease in Jiashi County of Xinjiang [16], with 1063 cases, accounting for 43.39% of the total cases reported between 2005 and 2010 (Table 1).

**Table 1:** Number of VL cases reported in China between 2005 and 2010

Year	AVL	MST-ZVL	DST-ZVL	Sub-total (Endemic areas)	Non-endemic areas	Total	%
2005	119	125	47	291	30	321	13.10
2006	91	139	31	261	26	287	11.71
2007	79	194	50	323	54	377	15.39
2008	63	179	253	495	29	524	21.39
2009	79	193	225	497	42	539	22.00
2010	75	180	102	357	45	402	16.41

Total	506	1010	708	2224	226	2450	100

### Geographic distribution

#### Differences in provinces

The 2450 cases were distributed at 179 counties/cities, in 18 provinces/municipalities/autonomous regions (Table 2). 97.71% (2394/2450) of cases occurred in three provinces/autonomous regions (namely Xinjiang, Gansu and Sichuan). The highest concentration (49.71%) of reported cases was in Xinjiang (n = 1218), followed by 33.67% in Gansu Province (n = 825), 14.33% in Sichuan Province (n = 351) (Table 3).

**Table 2:** VL cases distribution and endemic type between 2005 and 2010

		Type of endemic area		
		
County/city	No. of case		ZVL	
			
		AVL	MST-ZVL	DST-ZVL
Xinjiang				
Urumqi City	8	Yes		
Aksu City	8	Yes		
Wensu County	2	Yes		
Xayar County	11	Yes		
Wushi County	28	Yes		
Artux City	64	Yes		
Wuqia County	4	Yes		
Kashgar City	243	Yes		
Shufu County	88	Yes		
Shule County	19	Yes		
Yengisar County	13	Yes		
Shache County	17	Yes		
Xinhe County	1	yes		
Turpan City	3			Yes
Hami City	5			Yes
Korla City	22			Yes
Luntai County	16			Yes
Yuli County	21			Yes
Ruoqiang County	2			Yes
Zepu County Makit County	2			Yes
Yopurga County	5			Yes
Jiashi County	10			Yes
Bachu County	477			Yes
Minfeng County	39			Yes
Tumushuke city	2			Yes
Kuqa County	40			Yes
Karamay City	40			Yes
Toksun County	1			Yes
Yanji County	1			Yes
Baicheng County	1			Yes
Awat County	2			Yes
Moyu County	1			Yes
8 non-endemic counties/cities	19			
Gansu				
Wudou County	327		Yes	
Wenxian County	262		Yes	
Dangchang County	34		Yes	
Kangxian County	1		Yes	
Zhouchu County	112		Yes	
Diebu County	42		Yes	
Huanxian County	5		Yes	
Tongwei County	2		Yes	
Gangu County	3		Yes	
Linxia City	2		Yes	
Jiuqian City	1			Yes
Jinta County	3			Yes
Anxi County	1			Yes
Denhuang City	1			Yes
16 non-endemic counties/cities	28			
Sichuan				
Wenchuan County	10		Yes	
Lixian County	1		Yes	
Maoxian County	38		Yes	
Heishui County	47		Yes	
Beichuan County	4		Yes	
Jiuzhaigou County	107		Yes	
65 non-endemic counties/cities	144			
Shaanxi				
Yichuan County	1		Yes	
Shenmu County	1		Yes	
Qingjian County	1		Yes	
Ningqiang County	4		Yes	
6 non-endemic counties/cities	7			
Shanxi				
Yangquan County	3		Yes	
Xianghuan County	1		Yes	
Wuxiang	1		yes	
Inner Mongolia				
Ejni Banner	9			Yes
Other 12 non-endemic provinces				
23 non-endemic counties/cities	28			

*Total *	2450	13 counties/cities	23 counties/cities	25 counties/cities

**Table 3:** The distribution of VL cases in major provinces

Year	China NO. of case	Xinjiang NO. of case %		Gansu NO. of case %		Sichuan NO. of case %	
2005	321	165	51.40	92	28.66	57	17.76
2006	287	125	43.55	106	36.93	49	17.07
2007	377	129	34.22	160	42.44	75	19.89
2008	524	317	60.50	152	29.00	49	9.35
2009	539	304	56.40	161	29.87	62	11.50
2010	402	178	44.28	154	38.31	59	14.68

*Total*	2450	1218	49.71	825	33.67	351	14.33

#### Differences in endemic and non-endemic areas

**Figure 1:**
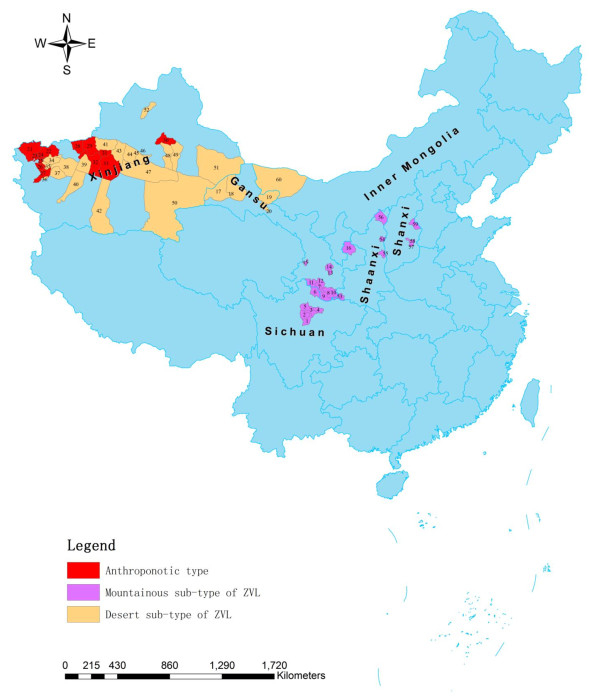
**The map of China indicating the distribution of VL endemic counties/cities in china between 2005 and 2010**. **Sichuan: **1. Wenchuan 2. Lixian 3. Maoxian 4. Beichuan 5. Heishui 6. Jiuzhaigou. **Gansu: **7. Zhouqu 8. Wudu 9. Wenxian 10. Kangxian 11. Diebu 12. Dangchang 13. Ggangu 14. Tongwei 15. Linxia 16. Huanxian 17. Dunhuang 18. Anxi 19. Jinta 20. Jiuquan. **Xinjiang: **21. Wuqia 22. Artux 23. Shufu 24. Kashgar 25. Shule 26. Yengisar 27. Shache 28. Wushi 29. Wensu 30. Urumqi 31. Xinhe 32. Aksu 33., Xayar 34. Jiashi 35. Yopurga 36. Zepu 37. Makit 38. Bachu (including Yumushuke City) 39. Awat 40. Moyu 41. Baicheng 42. Minfeng 43. Kuqa 44. Luntai 45. Korla 46. Yanqi 47. Yuli 48. Toksun 49. Turpan 50. Ruoqiang 51. Hami 52. Karamay. **Shaanxi: **53. Ningqiang 54. Qingjian 55. Yichuan 56. Shenmu. **Shanxi: **57. Xiangyuan 58. Wuxiang 59. Yangquan. **Inner Mongolia: **60. Ejni Banner.

Among 179 counties/cities, 61 counties/cities in six provinces/autonomous regions (33 in Xinjiang, 14 in Gansu, 6 in Sichuan, 4 in Shaanxi, 3 in Shanxi, and 1 in Inner Mongolia) were identified as VL endemic areas (Figure 1, Table 2) and reported 2224 autochthonous cases, accounting for 90.78% of the total reported cases in this period. The other 118 counties in 16 provinces/municipalities/autonomous regions were non-endemic areas and reported 226 (9.22%) imported cases (Table 2).

**Figure 2:**
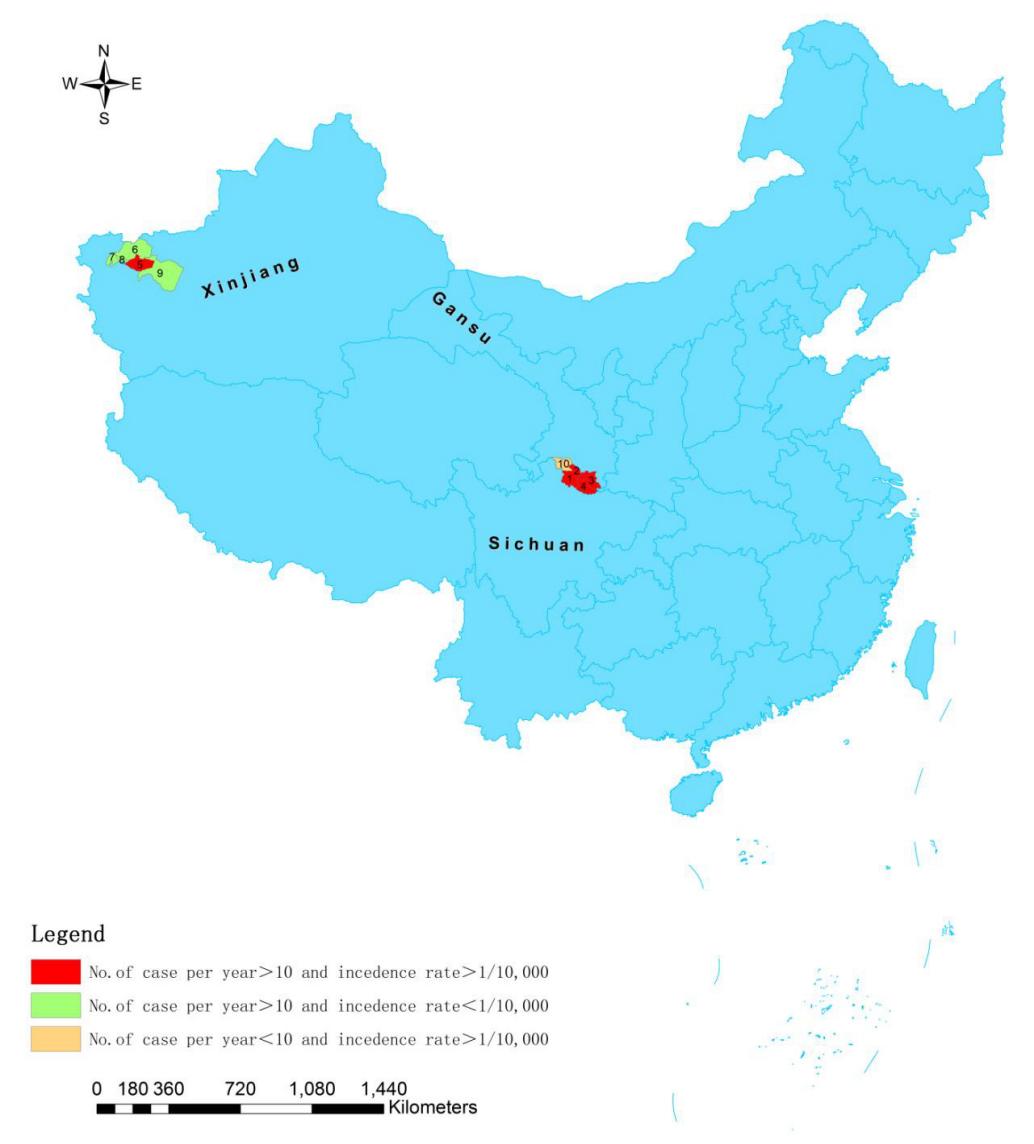
**The map of China indicating the major VL endemic counties/cities with a mean of more than 10 cases per year and/or with over 1/100,000 incidence rate**. 1. Jiuzhaigou 2. Zhouqu 3. Wudu 4. Wenxian 5. Jiashi 6. Artux 7. Shufu 8. Kashgar 9. Bachu 10. Diebu.

Out of 61 endemic counties/cities, 9 (14.75%) major counties/cities (including Artux City, Kashgar City, Shufu County, Jiashi County and Bachu County of Xinjiang, Wudou County, Wenxian County and Zhouchu County of Gansu, and Jiuzhaigou County of Sichuan) (Table 4, Figure 2) reported means of more than 10 cases per year with a total of 1759 cases reported, accounting for 79.09% (1759/2224) of total cases from endemic counties/cities and for 71.80% (1759/2450) of total cases reported in China in this period. 33 (54.10%) counties/cities reported means of less than 1 case annually. The other 19 (31.15%) counties/cities reported means of cases ≥ 1 and < 10 annually.

**Table 4:** No. of VL cases and incidence rate in major endemic counties/cities

counties/cities	No. of case	**Average No**.	Population**	Incidence rate
		of case per year		(1/100 000)
AVL				
Artux City	64	10.67	217,200	0.49
Kashgar City	243	40.5	427,200	0.95
Shufu County	88	14.67	303,200	0.48
Desert sub-type of ZVL				
Jiashi County	477	79.5	359,900	2.21
Bachu County (including Tumushuke city*)	79	13.17	447,400	0.29
Mountainous sub-typeof ZVL				
Wudou County	327	54.5	520,100	1.05
Wenxian County	262	43.83	238,500	1.84
Zhouchu County	112	18.67	135,100	1.38
Diebu County	42	7	53,400	1.31
Jiuzhaigou County	107	17.83	62,200	2.87

*Total*	1801	300.17	2,764,200	1.09

* Tumushuke city is located within Bachu County.

** 2007 census

Regarding the incidence rate, 7 counties/cities (including Jiashi County of Xinjiang, Wudou County, Wenxian County, Zhouchu County and Diebu County of Gansu, and Jiuzhaigou County of Sichuan) (Table 4, Figure 2) was over 1/100,000. The highest incidence rate (2.87/100,000) was in Jiuzhaigou County of Sichuan, followed by 2.21/100,000 in Jiashi of Xinjiang. The incidence rate in other counties was less than 2/100,000. The major endemic counties/cities with means of more than 10 cases per year or with over 1/100,000 incidence rate concentrated in two areas, namely, Kashi prefecture of Xinjiang and southern part of Gansu with adjacent Jiuzhaigou County of Sichuan (Figure 2).

In the period 2005 to 2010, 226 VL cases were reported from 118 non-endemic counties/cities in 16 provinces/municipalities/autonomous regions (Table 2). 222 cases were infected in China, and the other 4 cases were infected in Spain, Nigeria and Algeria, respectively.

#### Differences in natural environment

Among 61 endemic counties/cities, 13 counties/cities belong to an AVL endemic area and all concentrated in old oases in Kashi, Xinjiang, with 507 cases reported. 23 counties/cities (10 in Gansu, 6 in Sichuan, 4 in Shaanxi and 3 in Shanxi) belong to the MST-ZVL endemic area and are distributed in mountainous and hilly regions (including loess plateau), with 1009 cases reported. The other 35 counties/cities (20 in Xinjiang, 4 in Gansu and 1 in Inner Mongolia) are DST-ZVL and distributed in dry desert area and stony desert area, with 708 cases reported (Figure 1, Table 2).

### Demographic distribution

#### Age

The age distribution of cases is showed in Table 5. The majority of imported cases (87.17%) were in people of more than 14 years of age and the median age is 33.9 years. For the cases from endemic areas 44.65% occurred in the 0-2 years age group and the median age is 3.7 years. The age distribution of different types of VL was analyzed based on the case data of the major endemic counties/cities (including Artux, Kashgar, Shufu, Jiashi, Bachu, Wudou, Wenxian, Zhouchu, Diebu and Jiuzhaigou) listed in Table 4. For AVL, 43.04% occurred in the ≥ 15 years of age group with a median age of 12.1 years. For MST-ZVL, the percentages were 33.61%, 19.50%, 16.57%, and 30.32 in the 0-2, 3-6, 7-14 and ≥ 15 years age groups, respectively, and the median age is 6.1 years. For DST-ZVL the majority of cases (93.70%) occurred in the 0-2 years age group with the median age of 2.1 years.

**Table 5:** Age distribution of VL cases in each type of VL

Age (year)	Non-endemic area		Endemic area		AVL		MST-ZVL		DST-ZVL	
	No. of case	%	No. of case	%	No. of case	%	No. of case	%	No. of case	%
0-2	14	6.20	993	44.65	23	5.82	286	33.61	521	93.70
3-6	9	3.98	352	15.83	97	24.56	166	19.50	17	3.06
7-14	6	2.65	310	13.94	105	26.58	141	16.57	9	1.62
≥ 15	197	87.17	569	25.58	170	43.04	258	30.32	9	1.62
Total	226	100	2224	100	395	100	851	100	556	100

Median age	33.9		3.7		12.1		6.1		2.1	

#### Occupation

Regarding occupation, for imported cases labourers had the highest rate of VL infection (56.64%). In endemic areas infants and young children had the highest rate of infection (60.21%), followed by peasants (18.12%) and students (15.70%). The occupational distribution of different types of VL was analyzed based on the case data from the 9 major endemic counties/cities listed in Table 4. In AVL, students had the highest rate (33.67%), followed by infants and young children (29.62%), and peasants (29.62%). For MST-ZVL, the highest rate of cases was in infants and young children (52.41%), followed by peasants (22.54%) and students (18.57%). For DST-ZVL, most of cases were from infants and children (97.48%) (Table 6).

**Table 6:** Occupational distribution of VL cases

Group	Non-endemic area		Endemic area		AVL		MST-ZVL		DST-ZVL	
	No. of case	%	No. of case	%	No. of case	%	No. of case	%	No. of case	%
Infants and young children	24	10.62	1339	60.21	117	29.62	446	52.41	542	97.48
Students	9	3.98	349	15.70	133	33.67	158	18.57	5	0.90
Peasants	0	0	403	18.12	117	29.62	192	22.56	4	0.72
Labourers	128	56.64	19	0.85	0	0	8	0.94	1	0.18
Workers	15	6.64	20	0.90	3	0.76	10	1.18	0	0
Officials	8	3.54	35	1.57	6	1.52	17	2.00	2	0.36
Housewives	8	3.54	26	1.17	14	3.54	7	0.82	2	0.36
Personnel in service trades	6	2.65	5	0.22	0	0	4	0.47	0	0
Others	28	12.39	28	1.26	5	1.27	9	1.06	0	0

Total	226	100	2224	100	395	100	851	100	556	100

#### Gender

The gender distribution of cases is shown in Table 7. The ratio of males to females was 1:0.165, 1:0.678, 1:0.710, 1: 0.688, and 1:0.706 for non-endemic areas, endemic areas, AVL, MST-ZVL and DST-ZVL, respectively.

**Table 7:** Gender distribution of VL cases

Gender	Non-endemic area	Endemic area	AVL	MST-ZVL	DST-ZVL
male	194	1325	231	504	326

female	32	899	164	347	230

rate	1:0.165	1:0.678	1:0.710	0.688	0.706

### Temporal distribution

#### Year

In the period 2005-2010 the number of imported cases (from non-endemic areas) showed a slight difference year by year. While in endemic areas, reported cases increased from 2006, and reached the highest level in 2008 and 2009. Among them, reported cases increased year by year from 2005 to 2007 for MST-ZVL, and for DST-ZVL there was a sharp increase in 2008 and 2009 due to the outbreak occurring in Jiashi County of Xinjiang (Table 1).

#### Month

**Figure 3:**
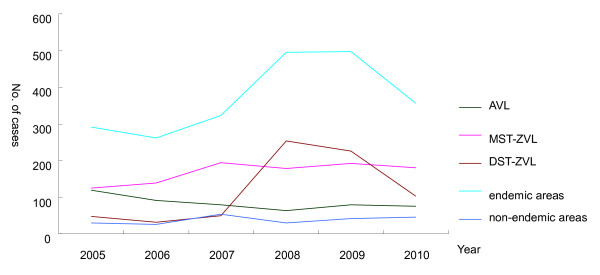
**Temporal (yearly) distribution of VL cases reported between 2005 and 2010**.

The monthly distribution of cases between 2005 and 2010 is shown in Figure 3. Cases were identified in all months for each type of VL, however, monthly distribution of reported cases showed a difference with different type of VL. For AVL the cases increased in March and reached a peak in June. The second small peak appeared in January. For MST-ZVL a peak occurred in April. However, for the DST-ZVL the majority of cases were reported in the period of September to February of the next year, with a peak in December.

## Discussion

The number of cases and endemic foci for VL has increased during the past two decades in the world [26-29]. A similar situation also occurred in China based on the results of this study. In the last six years a mean of 406 cases per year was reported with a 54.37% increase compared to a mean of 263 cases per year in the period of 1990-1999. 61 endemic counties/cities were identified with a 41.86% increase compared to 43 endemic counties/cities in the period of 1990-1999. In the last six years, endemic regions spread, such as Minfeng County of Xinjiang [23] or re-emerged, such as Gangu County of Gansu. Even an outbreak of the disease occurred in Jiashi County of Xinjiang in 2008-2009 with the highest incidence [16].

In China, different type or sub-type of VL (AVL, MST-ZVL and DST-ZVL) revealed differences not only in geographical and landscape characteristics, *Leishmania *species, vector species and reservoir host, but also in age and monthly distribution of cases [11,13,18,22,30,31]. Previous observation showed that for AVL 8.3-38.9% of the cases occurred under 5 year-old, with few infant cases. While for DST-ZVL, the majority (92%) of cases were aged < 2 years [19]. Present results indicate that 30.38 (5.82+24.56)% were in 0-6 years age group for AVL, 93.70% of cases of DST-ZVL were in 0-2 years age group, in agreement with previous observation. While alteration of the pattern of VL case age distribution was observed for MST-ZVL. Previous observation showed that 71.5-86.5% of cases were in the 0-5 years age group [19], the case data between 2005-2010 indicated that only 53.51 (33.61+19.50)% of cases were in the 0-6 years age group. The reason for a decreasing percentage of cases in infants and young children may be explained by the improvement of nutrition with the economic development or decreasing percentage of children in the total population because of family planning in China.

Sand fly season is from May to September in China. The monthly distribution of cases (Figure 3) showed that cases were identified in all months for AVL and MST-ZVL, while the cases were clustered in the period of September to February of next year for DST-ZVL, indicating that the incubation period of *Leishmania *infection is shorter in DST-ZVL than in AVL and MST-ZVL, consistent with the highest percentage of infantile cases with the state of relative immaturity of cellular immunity.

The present study showed a male predominance among cases, similarly to what has been reported by Willian DSN, et al [32]. We have to investigate whether gender was a risk factor or the result was due to maladjustment of gender in population in China. The reason for the higher susceptibility presented by infants and young children could be explained by the state of relative immaturity of cellular immunity.

**Figure 4:**
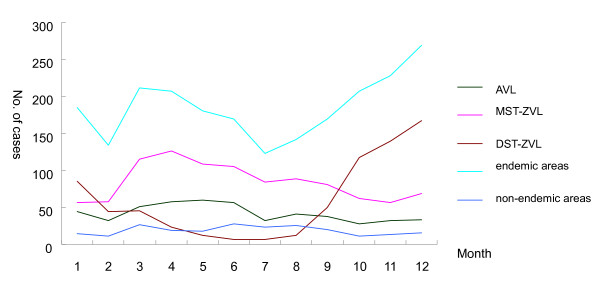
**Temporal (monthly) distribution of VL cases reported between 2005 and 2010**.

Kashgar city of Xinjiang is an AVL endemic area with peridomestic *P. longiductus *as the vector. From 2000, VL cases increased year by year (Figure 4). With the implementing of control measure (spraying of insecticides to the house of VL patients and their neighbor) beginning in 2003, cases dropped stepwise (Figure 4). This fact indicated that spraying of insecticides is an effective measure for control of AVL in China. Jiuzhaigou County of Sichuan is a MST-ZVL endemic area with half of dogs infected by *L. infantum *[22]. Considering the county is a famous tourism resort, the local authorities advocated killing of dogs beginning in 2005, with a cumulative decrease of half the canine population. Accordingly, the number of human VL case dropped from 28 cases in 2006 to 7 cases in 2010 (Figure 4). Therefore, Control measures, such as treatment or eradication of infected dogs, or prohibition of maintaining dogs, must be taken against MST-ZVL.

The desert sub-type is unusual and endemic in the northwestern desert regions of China, including Xinjiang, western Inner Mongolia and northern Gansu, where it is considered to be a natural nidus of kala-azar infected wild animals presumably being the source of infection and where the wild species, *P. wui *and *P. alexandri*, are the vectors [19,23]. An outbreak of VL in the period of 2008 to 2009 in Jiashi County of Xinjiang, a DST-ZVL endemic area, with the incidence rate increasing more than twenty-fold compared to the average annual incidence (Figure 4). After an outbreak occurred we initiated an active surveillance and found that all cases were autochthonous and more than 90% of cases occurred in infants, while the majority of anthroponotic cases occur among young adults. Most desert sub-type kala-azar cases occur between October and December, whereas the peak of case onset for the anthroponotic type is from April to May, with a secondary peak from September to October. The causative agent for DST-ZVL was identified as *L. infantum *whose hosts are animals, while little dogs (main reservoir host for ZVL) were found in the outbreak area [16]. We conclude that DST-ZVL is an unusual ZVL. Surprisingly, the number of cases dropped to a normal level in 2010 (only 7 cases were reported after the sandfly season in 2010) with no measures to implement (the vector is wild and reservoir host is unknown). The situation is similar to that which occurred in the Kzyl-Orda region of the Kazakhstan reported by Genis who stated "the dynamics of the incidence is of a 'pulsating' nature and is determined by changes in the status of natural foci of visceral leishmaniasis" [33]. The identification of the reservoir host and study of a control strategy is essential against DST-ZVL in China.

Global warming, globalisation, and the constantly increasing numbers of people involved in long-distance tourism and travel to exotic destinations are likely to increase the number of cases of exotic diseases "imported" to non-endemic countries. The recent literature indicates a sharp increase in imported leishmaniasis cases in developed, non-endemic countries over the last decade, in association with increasing international tourism, military operations, and the influx of immigrants from endemic countries [10,34-37]. In China, imported cases have become a important public health problem with a mean of 40 reported cases per year, and most of cases of exotic VL imported to non-endemic areas was by male labourers on short-term work in endemic areas. Therefore, these people are at particular risk. Appropriate counseling should be provided to the people likely to be exposed to sandflies in endemic areas.

## Conclusions

The results from the present study indicate that the number of visceral leishmaniasis cases and of endemic counties both increased in the period 2005-2010 in China compared to that of the 1990s. Differential control measures must be taken in different endemic areas against incidence increase and endemic area spread.

## Competing interests

The authors declare that they have no competing interests.

## Authors' contributions

JYW designed and conducted the study, performed data collection/analysis and drafted the manuscript. GC and HTC performed data collection/statistical analysis. HNZ outlined the draft. CHG and YTY drew the tables and figures. All authors read and approved the final manuscript.
